# Cochlear duct length variability in prelingual sensorineural hearing loss: Emphasis on Waardenburg syndrome and implications for cochlear implantation

**DOI:** 10.1007/s00405-026-10046-w

**Published:** 2026-03-05

**Authors:** Kyu Ha Shin, Chang-Hee Kim, Yehree Kim, Jung Kyu Lee, Hye-Rim Park, Jin Hee Han, Jiyeon Yang, Bong Jik Kim, Byung Yoon Choi

**Affiliations:** 1 Department of Otorhinolaryngology-Head and Neck Surgery, Seoul National University College of Medicine, Seoul National University Bundang Hospital, Seongnam, 13620 Republic of Korea; 2 Sensory Organ Research Institute, Seoul National University Medical Research Center, Seoul, South Korea; 3 Department of Otorhinolaryngology-Head and Neck Surgery, Konkuk University Medical Center, Research Institute of Medical Science, Konkuk University School of Medicine, Seoul, Republic of Korea; 4 Climate-Environment-Society-Digital Innovation (CSI) Lab, Chungnam National University, Daejeon, Republic of Korea

**Keywords:** Cochlear duct length, Etiology, Cochlear implant, Angular insertion depth, Waardenburg syndrome

## Abstract

**Purpose:**

To explore the association between cochlear duct length (CDL) and hearing loss etiologies, and evaluate its impact on cochlear implantation (CI) planning.

**Methods:**

We conducted a retrospective study on 610 patients (825 ears) who underwent CI at a tertiary center, to identify the etiologies associated with reduced CDL and investigate their influence on electrode selection and surgical outcomes.

**Results:**

Patients diagnosed with cochlear nerve deficiency, Waardenburg syndrome (WS) associated with *SOX10* variants, enlarged vestibular aqueduct syndrome, and incomplete partition type III exhibited significantly reduced CDL. Of these, WS associated with *SOX10* variants exhibited the highest decrease in CDL, often with vestibular malformations.

**Conclusions:**

Short CDL poses surgical challenges, necessitating tailored electrode selection for optimal CI outcomes. In *SOX10*-related WS cases, preoperative imaging and genetic testing can aid in the early detection of the disease, enabling appropriate CI planning. These findings indicate the importance of having a preliminary estimation of CDL based on etiology and the significance of this study.

## Introduction

In cochlear implantation (CI), accurate estimation of cochlear duct length (CDL) is crucial for selecting the appropriate electrode and determining the optimal insertion depth [1–3]. Given the advancements in residual hearing preservation techniques, meticulous insertion of electrodes to a specific depth, which prevents damage to residual hearing, is of great importance [4–7]. Especially in cases involving less-experienced surgeons or rapid electrode insertions, tactile feedback is diminished or lost, significantly increasing the risk of unintended deep insertional trauma [8, 9]. Furthermore, numerous studies have reported a correlation between the angular insertion depth of CI electrodes and postoperative speech outcomes [10–18]. Although the impact of deeper insertion on speech outcomes may differ between lateral wall arrays [10–14] and pre-curved arrays [15–18], this relationship remains an important consideration. Therefore, it is crucial to employ a preoperative technique that enables correct estimation of the CDL.

In the field of CI, accurately determining the CDL to optimize electrode insertion depth in anatomically normal cochleae has been extensively studied [19, 20]. Specifically, in 2005, Bernard Escudé, a radiologist from France, introduced a method using the diameter of the basal turn (referred to as the "A-value"), measured in an oblique coronal plane commonly known as the "cochlear view" [20]. He proposed that this parameter could reliably estimate the CDL along the outer lateral wall, extending from the round window entrance to a specified insertion depth. Notably, inner ear malformations (IEMs) have been globally documented in approximately 20%–30% of pediatric patients presenting with congenital hearing impairment [21–23]. Although cochleae with abnormal anatomy have been extensively investigated using two-dimensional radiographic imaging [21–23], the existing A-value approach utilized for CDL estimation does not adequately account for the structural complexities found in malformed cochleae. Consequently, conventional CDL equations are not applicable to structurally abnormal cochleae. Instead, capturing the complete external morphology of severely malformed inner-ear structures using three-dimensional (3D) imaging techniques is essential [24–26]. Thus, outer wall length (OWL) or lateral wall length measurements based on 3D reconstructions become critical. Alternatively, the OTOPLAN® software (CAScination AG, Bern, Switzerland, in collaboration with MED-EL, Innsbruck, Austria), which is currently the only CE-certified DICOM viewer for CI, offers an advanced solution. This software allows comprehensive management of pre-, intra-, and postoperative imaging, precise CDL estimation, and individualized frequency mapping [27–30], making it suitable for evaluating cochleae even with anatomical malformations such as enlarged vestibular aqueduct (EVA), incomplete partition type I, II and III (IP-I, II, and III) [31–34].

In cases of clear IEMs, reductions in OWL or A-value have been consistently reported compared to normal cochleae. However, CDL characteristics associated with etiologies involving molecular-genetic factors or other forms of hearing loss unrelated to direct structural cochlear malformations remain largely unexplored. The CDL in normal cochleae has been shown to vary according to gender, laterality, and ethnicity [35–37]. Although conventional CDL estimation equations are generally inadequate for the cochleae from IEMs, recent studies indicate that advanced 3D imaging platforms, such as OTOPLAN®, enable precise morphological assessments even when only a partial modiolus is present. Thus, utilizing the 3D-based OTOPLAN® software, we aimed to evaluate and compare CDL measurements across diverse congenital hearing loss etiologies. These include conditions previously assessed by OTOPLAN®, such as IP- I and IP- III and EVA [27–30], as well as conditions not yet systematically studied or established in terms of cochlear size, including cochlear nerve deficiency (CND), Waardenburg syndrome (WS), and congenital cytomegalovirus (cCMV)-related hearing impairment.

Estimation of CDL is especially critical in cases with significantly short CDL, defined statistically as greater than two standard deviations below the mean, because the risks associated with unintended deep electrode insertion are significantly heightened in such cases. This can result in basilar membrane damage, electrode misplacement into the scala vestibuli, or extension of the basal end of the electrode array beyond the cochlear boundaries. The complications arising from a short CDL are associated with not only the use of slim straight electrodes but also slim modiolar electrodes (SMEs). In the latter case, in addition to the aforementioned issues, excessive insertion may reduce modiolar proximity, thereby worsening interelectrode interference and potentially compromising the auditory performance [18].

However, routine CDL measurement may not always be feasible at CI centers, and decisions regarding electrode selection and insertion depth often must be made during preoperative consultations prior to obtaining precise CDL measurements. In anticipation of such clinical circumstances, it would be highly beneficial to identify and be aware of specific etiologies linked to a significantly short CDL, as these cases are particularly vulnerable to the adverse consequences associated with unintended deep insertion. Therefore, in this study, we sought to evaluate the CDL in pediatric patients with severe-to-profound hearing loss, using the OTOPLAN® software tool, with a specific focus on identifying etiologies associated with significantly short CDL. Preoperative imaging, including TBCT, plays a critical role in CI surgical planning, and measurement of the CDL using such imaging provides essential information for appropriate electrode selection. Hence, preoperative assessment of CDL is strongly recommended whenever possible. However, in cases where CDL measurement is not feasible due to institutional limitations, the findings of this study may offer valuable guidance for preoperative CI surgery planning.

## Patients and methods

### Subjects

We recruited a large cohort of patients who underwent CI from May 2020 to December 2023 due to severe-to-profound hearing loss in a single tertiary center. This study involved 610 patients (825 ears) including 440 adults (534 ears) and 170 young children (291 ears), with the latter defined as those aged 2 years or below (Table 1). Whenever possible, genetic testing, imaging examinations, and urine tests for congenital cytomegalovirus (cCMV) were conducted.

**Table 1:** Number of patients (ears) included in the study

All age	Under 2 years old
610 patients (825 ears)	170 patients (291 ears)

### Measurement of cochlear duct length

**Fig. 1:**
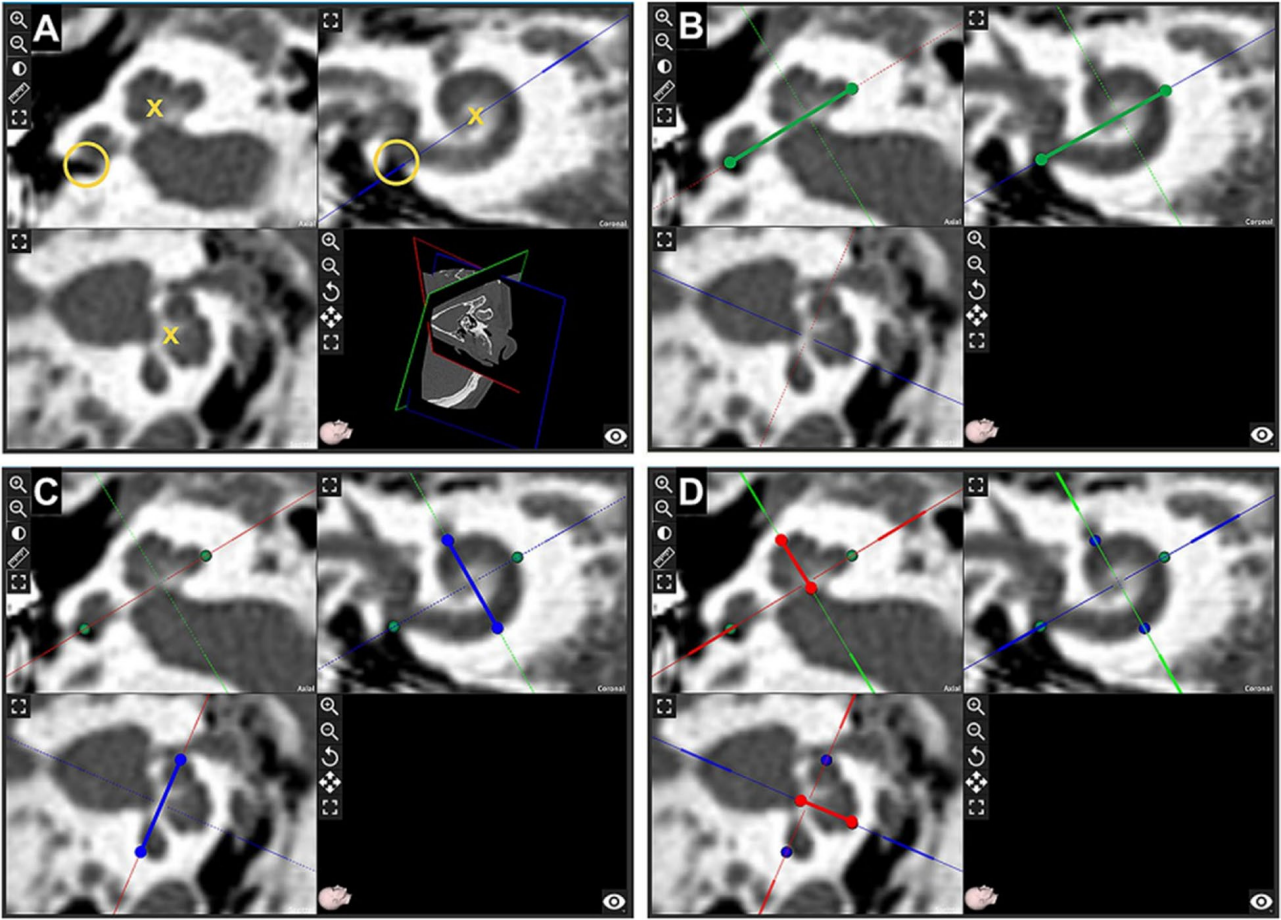
Steps of CDL measurement using the OTOPLAN software. **A** Multiplanar reformation of the right inner ear reconstructed along the basal cochlear turn. The center of the modiolus (yellow cross) and the round window (yellow empty circle) are visible. **B** A value is measured as the distance between the round window and the contralateral cochlear wall: solid green line connecting two green dots. **C** B value represents the cochlear width perpendicular to the A value: solid blue line connecting two blue dots. **D** The height of the cochlea is measured on a plane orthogonal to the basal turn of the cochlea: solid red line connecting two red dots

Before CI, CDL was assessed in all 610 patients (825 ears) using the OTOPLAN software (CAScination, Bern, Switzerland) (Fig. 1) [38]. The OTOPLAN offers both manual and automated calculation methods [39, 40], and the manual measurement approach was employed in the present study. DICOM files from preoperative computed tomography images (slice thickness, 0.75 mm) were imported into the OTOPLAN software, and the data were independently analyzed by two otolaryngologists (K.H.S and B.Y.C) via multiplanar reformation. An image plane parallel to the basal turn of the cochlea was reconstructed, and “diameter, A value” (the largest distance from the round window [RW] to the contralateral wall) and “width, B value” (the distance between the cochlear walls perpendicular to the diameter line) were measured. The cochlear height was measured in an orthogonal plane (H value). Utilizing the determined values (diameter, width, and height), the CDL was calculated using the OTOPLAN software, employing the elliptic–circular approximation (ECA) method [41]. The ECA is a geometrical method used in OTOPLAN to estimate CDL by modeling the basal turn as a half-ellipse plus a half-circle derived from patient-specific A and B value measurements on TBCT. The basal-turn length is scaled to the angular insertion depth to yield CDL, typically reported along the lateral wall or along the Organ of Corti for frequency-place planning, and this approach improves accuracy over older A value-only prediction models. A four-step method was employed to measure cochlear parameters and calculate the CDL. TBCT axial images were imported into OTOPLAN, and coronal and sagittal views were reconstructed using MPR planes to establish the cochlear coordinate system. Six cursors were then placed at predefined anatomical landmarks, including the round window and lateral wall (A value), lateral wall inferiorly and lateral wall superiorly (B value), and cochlear base and cochlear apex (H value), within the software interface. The software subsequently generated the CDL and its related parameters automatically.

We obtained the overall CDL distribution and further analyzed the distribution of specific conditions with a significant number of cases. We first determined the overall CDL distribution and defined a significantly shortened CDL, termed “short CDL”, as any CDL shorter than −2 standard deviations (SD). Determination of short CDL was made based on the distribution derived from our study cohort. A CDL shorter than − 2 SD was used as the cut-off because, assuming a normal distribution of CDL, the typical range encompasses approximately 95% of the population when ± 2 SD is adopted as the threshold. Then, we identified the etiologies that presented with short CDL in at least two ears. Subsequently, we established a separate cohort exclusively consisting of patients exhibiting the identified etiologies and designated it as the “short CDL group”. We compared the mean values of CDL measurements of the entire group and each short CDL subgroup, determining whether the probability of short CDL in each etiology subgroup was higher than that in the total patient population. The analysis was repeated, with a focus on the categorization of patients below 2 years old.

An independent t-test or Mann–Whitney test was used to assess statistical significance in continuous variables, and the Fisher’s exact test was used to evaluate the probability of short CDL among patients with each subgroup etiology being significantly higher than the probability of short CDL in the total patient population or in individuals aged 2 years or below. A *p*-value of < 0.05 was considered statistically significant. All statistical analyses were performed using SPSS Statistics for Windows, version 19.0 (IBM Corp., Armonk, NY, USA).

## Results

**Fig. 2:**
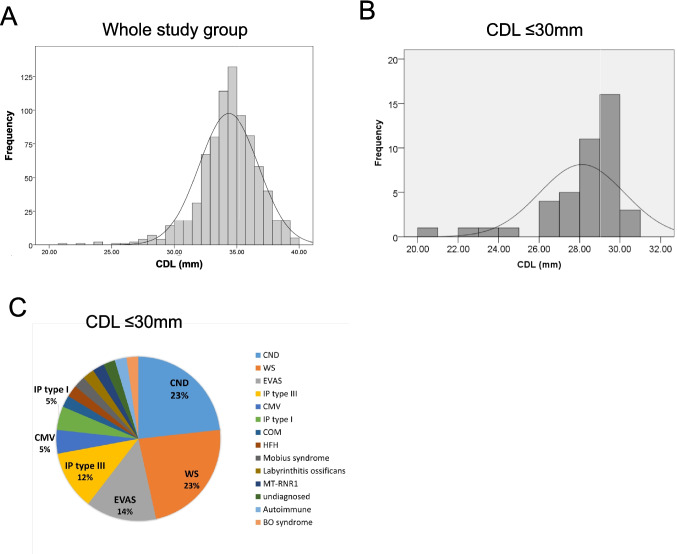
**A** Histogram with a normal distribution curve of the overall cochlear duct length (CDL) measured using OTOPLAN ver. 3.0 in whole study group (*n* = 825). The average, maximum, and minimum CDLs were 34.36, 39.80 and 20.80 mm, respectively. **B** Histogram with a normal distribution curve of the short CDL group (CDL ≤ 30 mm) measured using OTOPLAN ver. 3.0 (*n* = 43). The average, maximum, and minimum CDLs were 28.15, 30.60 and 20.80 mm, respectively. **C** Etiology of the group with CDL ≤ 30 mm. CND, cochlear nerve deficiency; WS, Waardenburg syndrome; EVAS, enlarged vestibular aqueduct syndrome; IP type III, incomplete partition type III; cCMV, congenital cytomegalovirus infection; IP type I, incomplete partition type I; COM, chronic otitis media; HFH, high-frequency hearing loss; MT-RNR1, mitochondrial 12S ribosomal RNA gene mutation; BO syndrome, branchio-otic syndrome

The CDL distribution in the 610 patients (825 ears) was determined, expressed as mean and SD (Fig. 2A). A total of 43 ears had a CDL shorter than 30 mm, corresponding to −2 SD (Fig. 2B), and the identified etiologies of 43 short CDLs were as follows; CND (*n* = 10), Waardenburg syndrome (WS, *n* = 10), enlarged vestibular aqueduct syndrome (EVAS, *n* = 6), congenital cytomegalovirus infection (cCMV, *n* = 2), incomplete partition (IP) type III (*n* = 5), IP type I (*n* = 2). chronic otitis media (COM, *n* = 1), high-frequency hearing loss (HFH, *n* = 1), Mobius syndrome (*n* = 1), labyrinthitis ossificans (*n* = 1), mitochondrial 12S ribosomal RNA gene mutation (MT-RNR1, *n* = 1), autoimmune inner ear disease (*n* = 1), branchio-otic syndrome (BO syndrome, *n* = 1) and unknown etiology (*n* = 1) (Fig. 2C). Since only etiologies with two or more cases were decided to analyze, a total of 35 ears (15 right ears and 20 left ears) with short CDL were included in the study. The short CDL group, with at least two cochleae with short duct length (< 30 mm), consisted of patients with CND, EVAS, WS, cCMV, IP type III, and IP type I. A total of 8 cases, each with only one case showing short CDL (including chronic otitis media, high jugular bulb-related hearing loss, Möbius syndrome, labyrinthitis ossificans, MT-RNR1 variant, autoimmune inner ear disease, BO syndrome, and unknown etiology), were excluded from the analysis. (Table 2).

**Table 2:** Relationship between cochlear duct length (CDL) and causes of hearing loss in patients of all ages and those aged 2 years or below

Etiology	Number of ears		Short CDL cases (< 30 mm)		Average CDL^†^	*P*-value for the probability of short CDL cases^‡^	
	All age	Under 2 years old	All age	Under 2 years old	All age	All age	Under 2 years old
CND	77	61	10	9	33.47 ± 2.66, ^*^p = 0.016	^*^p < 0.001	*p* = 0.057
WS	18	18	10	10	30.13 ± 2.39, ^*^p = 0.001	^*^p < 0.001	^*^p < 0.001
cCMV	30	21	2	1	34.74 ± 3.24, *p* = 0.412	*p* = 0.741	*p* = 0.541
EVAS	58	0	6	0	33.12 ± 2.09, ^*^p = 0.001	*p* = 0.056	N/A
IP III	10	4	5	2	29.15 ± 2.07, ^*^p = 0.002	^*^p < 0.001	^*^p = 0.025
IP I	8	7	2	1	34.35 ± 2.36, *p* = 0.167	*p* = 0.063	*p* = 0.459

*CND* Cochlear nerve deficiency; *WS* Waardenburg syndrome; *cCMV* Congenital cytomegalovirus; *EVAS* Enlarged vestibular aqueduct syndrome; *IP* Incomplete partition; N/A, not applicable. ^*^A *P*-value < 0.05 was considered statistically significant. ^†^A *P*-value indicates whether each etiology-specific group shows a statistically significant difference in CDL compared with the CDL from the total cohort. ^‡^A *P*-value here represents the probability of short CDL among patients with each subgroup etiology being significantly higher than the probability of short CDL in the total patient population or in individuals aged 2 years or below (Fisher's exact test)

In the short CDL group, the mean CDL of the CND (*p* = 0.016), WS (*p* = 0.001), EVAS (*p* = 0.001), and IP type III (*p* = 0.002) groups was significantly shorter compared with the overall cohort. From a slightly different analytical perspective, the patients with CND, WS, and IP type III exhibited a higher tendency of having a CDL ≤ 30 mm compared with the probability of a randomly selected ear from the overall cohort having a CDL of ≤ 30 mm (Table 2). When the analysis was limited to patients aged below 2 years, those with WS and IP type III still exhibited a higher probability of having a CDL ≤ 30 mm (Table 2). Among the patients with etiologies associated with short CDL, those with CND and WS were implanted with Cochlear^©^ SME, whereas those with IP type III were implanted with Cochlear^©^ 24RE-ST full-band straight electrode. In CND, CI was performed for cases of bilateral sensorineural hearing loss.

Before the analysis, we did not anticipate that the WS cases would have such a significantly shorter CDL than the average, although a small cochlea had been reported in the literature as being associated with WS caused by *SOX10* variants [42]. Therefore, we conducted a more comprehensive investigation of the cochlea from nine WS cases (Table 3), of which eight were classified as WS2 and one as WS4. Notably, eight (88.8%) of the nine WS cases were found to have pathogenic *SOX10* variants. One patient with WS2 had an *MITF* variant. Interestingly, unlike patients with *SOX10* variants, this patient exhibited no vestibular malformation, and their CDL fell within the normal range (Tables 2 and 3). Therefore, the association between WS and short CDL in this study appears to be mainly linked to the relationship between *SOX10* variants and short CDL (Table 3). Notably, among eight WS patients with short CDL, seven demonstrated vestibular malformation, as evidenced by imaging modalities, and WS occurred de novo in six out of nine families without any family history (Table 3).

**Table 3:** Causative genes and cochlear duct length (CDL) in patients with Waardenburg syndrome (WS)

Proband ID (Gender/Age at visit)	Variant cDNA/protein change	dbSNP ID [Build v156]	Inheritance	Characteristics		ACMG/AMP guideline				Reference (PMID)
				Clinical features	CDL (mm)	Criteria	Classification			
*SOX10* [NM_006941.4]:[NP_008872.1]										
SB787-1376 (F/3mon)	c.1085_1086dup:p.Pro363Gly*fs*Ter140, *SOX10*	Absent	AD	WS2 VM	28.3/27.9	PVS1, PS2_Moderate, PM2, PP4		Pathogenic		This study
SB1237-1978 (M/7mon)	c.717dup:p.Thr240His*fs*Ter41,* SOX10*	Absent	AD, Sporadic	WS2	28.4/26.2	PVS1, PS2_Moderate, PM2, PP4		Pathogenic		This study
SB304-606 (M/2)	exon4 deletion (pGly233_Pro466del?), *SOX10* based on MLPA	Absent	AD, Sporadic	WS4 VM	30.9/31.5	PVS1, PS2_Moderate, PM2, PP4		Pathogenic		This study
SB1326-2090 (F/8mon)	c.698-5C > G,* SOX10*	rs779080291	AD, Sporadic	WS2 VM	32.6/32.9	PVS1, PM2, PP3, PP4		Pathogenic		This study
SB958-1605 (F/2mon)	c.610C > T:p.Gln204Ter,* SOX10*	rs2145768136	AD, Sporadic	WS2 VM	30.4/30.3	PVS1, PS2_Moderate, PM2, PP4		Pathogenic		This study
SB690-1235 (F/3mon)	c.424 T > C:p.Trp142Arg,* SOX10*	rs1555939408	AD	WS2 VM	28/29.4	PS1, PS2_Moderate, PM1, PM2, PP3, PP4		Pathogenic		PMID: 23643381, 24033266, 29453417 This study,
SB1306-2064 (F/8mon)	c.160_190dup:p.Asp64Gly*fs*Ter13,* SOX10*	Absent	AD, Sporadic	WS2 VM	29.2/29.6	PVS1, PS2_Moderate, PM2, PP4		Pathogenic		This study
SB1220-1961 (F/3mon)	c.−84-2A > C,* SOX10*	Absent	AD, Sporadic	WS2 VM	28.9/28.2	PVS1, PS2_Moderate, PM2, PP4		Pathogenic		PMID: 27938609, This study
*MITF* [NM_000248.3]:[NP_000239]										
SB1358-2131 (M/41mon)	c.33 + 1G > T, *MITF*	Absent	AD	WS2	34.33/35 15	PVS1, PM2, PP3, PP4		Pathogenic	This study	

*M* Male; *F* Female; *HGVS* Human genome variation society; *MLPA* Multiplex ligation-dependent probe amplification; *dbSNP* Single nucleotide polymorphism database; *AD* Autosomal dominant; *VM* Vestibular malformation; *ACMG* The American College of Medical Genetics and Genomics; *AMP* The Association for Molecular Pathology; *PVS* Pathogenic very strong; *PS* Pathogenic strong; *PM* Pathogenic moderate; *PP* Pathogenic supporting; *PMID* PubMed IDentifier

## Discussion

There are numerous reports in the literature on CDL measurement in pediatric CI recipients. However, studies that explicitly delineated the differences in CDL based on the etiology of CI recipients remain limited [1, 43–45]. In this context, we analyzed CDL variations across different etiologies in a large cohort of CI recipients from a tertiary center. We showed that patients with CND, WS, and EVAS with/without IP types II and III exhibited significantly shorter CDL. Notably, patients with a CDL ≤ 30 mm are more likely to face challenges in achieving optimal electrode positioning and configuration during CI. In our cohort, CND, WS, and IP type III fell into this category, indicating the potential surgical difficulties associated with these conditions.

Among WS, IP type III, CND, and EVAS, EVAS and IP type III were previously reported to have a shortened *A value* [26]; thus, these findings did not warrant particular attention in the analysis. However, CND and WS cases were not expected to exhibit significantly shorter CDL prior to the study initiation, which makes these findings particularly intriguing.

Cochlear duct elongation is regulated by neurotrophin-independent signaling pathways, such as fibroblast growth factor (FGF) and sonic hedgehog (SHH), and neurotrophin-dependent mechanisms, including brain-derived neurotrophic factor (BDNF), neurotrophin-3 (NT-3), and extracellular matrix (ECM) remodeling [46]. In patients with CND, the absence or hypoplasia of the cochlear nerve results in a reduction in only neurotrophin-dependent signaling, which could partially restrict cochlear duct elongation. This diminished neurotrophic support may contribute to a statistically significant, albeit not critical, reduction in CDL compared with the other patients in our cohort. To validate this hypothesis, it is essential to investigate the correlation between CDL and CND severity. Given the need for a large sample size, we are preparing this research topic as a follow-up study.

Understanding the intricate relationship between CDL, electrode insertion depth, and the preservation of residual hearing is important for optimizing patient outcomes in CI. Individual variability in CDL necessitates careful selection of electrode and insertion depth to mitigate intracochlear trauma [1]. Research indicates that while deeper electrode insertion may improve frequency mapping [47], it concurrently elevates the risk of residual hearing loss due to potential mechanical disruption of delicate cochlear structures [48]. Conversely, utilizing shorter electrode arrays may help preserve residual hearing but could restrict access to lower-frequency sounds [49]. This delicate balance between insertion depth and hearing preservation is especially pertinent for patients possessing functional low-frequency hearing, where atraumatic surgical techniques and personalized electrode selection are paramount [50].

In this study, we also investigated the CDL in WS and explored the causative genes, clinical phenotypes, and their correlations as well as how these can be used in CI. One of the most compelling findings of this study is that WS exhibited the highest likelihood of a short CDL among all etiologies. Notably, all WS cases found to have a short CDL were associated with *SOX10* variants. However, the CDL of one WS2 patient with an *MITF v*ariant was not shortened. Some patients with unilateral or asymmetric hearing loss were also diagnosed with WS associated with *MITF* or *PAX3* variants. However, they were not candidates for CI and were thus excluded from the study. The exceptionally short CDL observed in *SOX10*-related WS cases in this study might not be entirely surprising based on the previous study, which reported that *SOX10*-related WS was associated with small and flattened cochlear morphologies [42]. From a developmental perspective, the greater impact of *SOX10* variants on CDL than CND could be attributed to the role of *SOX10* in modulating neural crest cell function [51]. Specifically, while the absence of the cochlear nerve in CND does not directly impair cochlear duct morphogenesis, *SOX10* alteration introduces additional perturbations, particularly affecting glial cell function and ECM dynamics [52], which ultimately results in a more pronounced cochlear duct shortening. In other words, unlike *SOX10* variants, which impair neural crest-derived glial cell function and neurotrophic signaling, CND mainly affects neuronal presence, leading to a less-pronounced impact on cochlear duct size than *SOX10*-related WS cases.

**Fig. 3:**
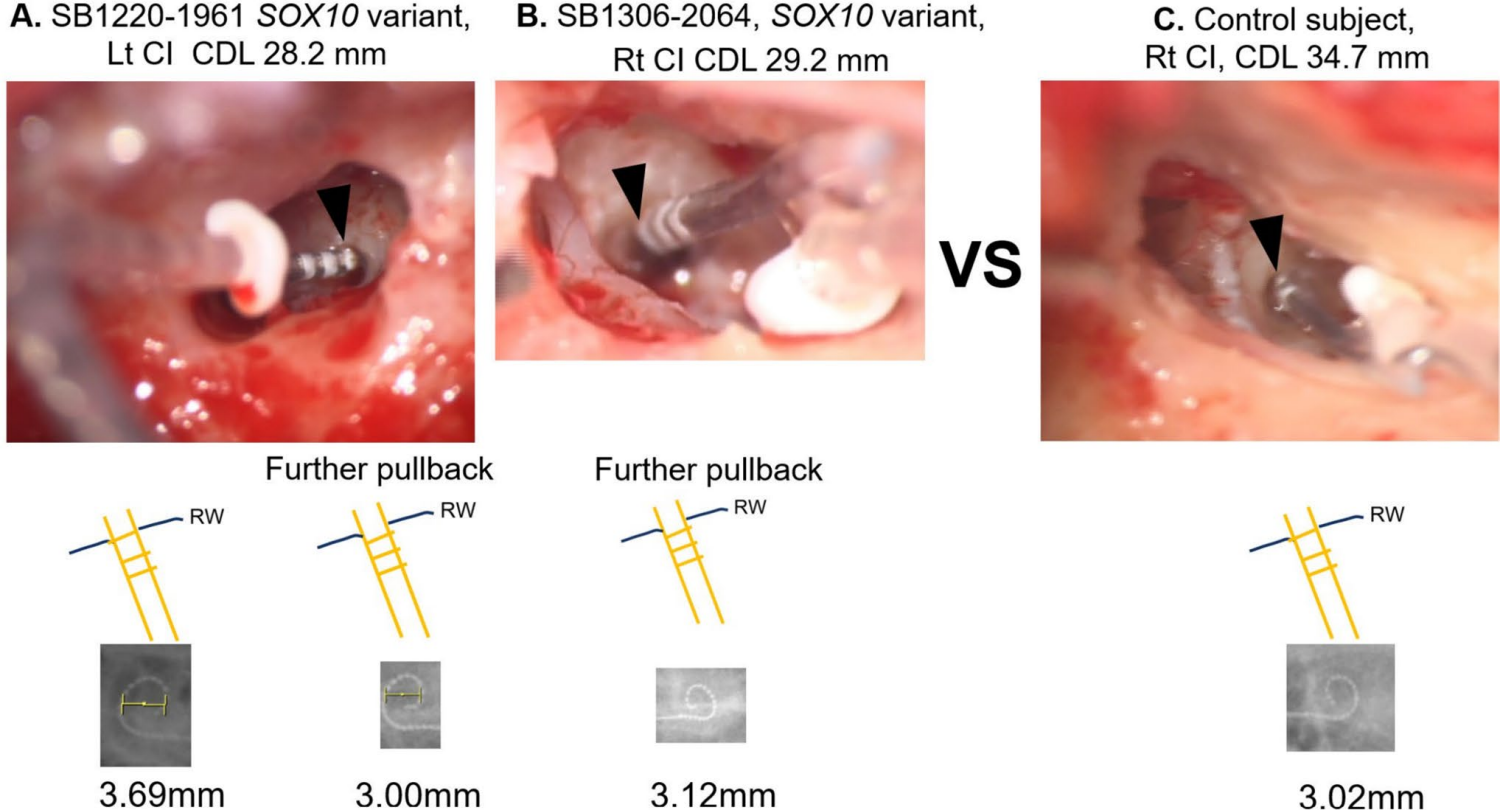
Comparison of the insertion depth of the slim modiolar electrode (SME) between the short cochlear duct length (CDL) and the average CDL. **A**, **B** SME insertion findings in a *SOX10*-related WS2 patient with a short CDL. The electrode was secured by applying the further pullback method, positioning the most distal marker (black arrowhead) slightly outside the round window membrane (RWM) (bottom right of A and bottom of B). As observed on the intraoperative X-ray, this approach improves modiolar proximity compared with the conventional insertion depth, where the most distal marker is positioned at the RWM level (bottom left of A). **C** SME placement in a cochlea with average duct length. In this case, optimal modiolar proximity is naturally achieved at the standard insertion depth, where the most distal marker (black arrowhead) aligns with the RWM level (bottom of C). The X-ray-based spiral diameters were 3.69, 3.00, and 3.12 mm, respectively, with a smaller spiral diameter indicating better modiolar proximity, as suggested by Lee et al. [54]

The identification of short CDL in CND and *SOX10*-related WS cases in our study holds clinically significant implications, particularly in the context of CI. In bilateral profound hearing loss associated with WS, the presence of vestibular anomalies on imaging strongly indicates the likelihood of an underlying *SOX10* variant. In such cases, lateral straight electrodes may be preferred if substantial residual hearing is preserved. Even when CDL measurement is unavailable, it can be reasonably anticipated that CDL ≤ 30 mm, allowing for informed preoperative electrode selection. Conversely, an SME may be more suitable for cases of bilateral total profound deafness, where residual hearing preservation is not a concern and achieving optimal modiolar proximity is prioritized. It was reported that modiolar proximity is influenced by the insertion depth [18], indicating that adjustment of the insertion depth can enhance electrode positioning relative to the modiolus. Therefore, in cases where a short CDL is expected, a further pullback strategy may be beneficial. Specifically, insertion of the electrode slightly shallower than usual—so that the most distal white marker remains just outside the RW—can improve modiolar proximity (Fig. 3). This consideration is particularly crucial for patients with CND, as better modiolar proximity is thought to likely improve ECAP responses and yield better behavioral outcomes [53]. As CND requires a greater degree of electrical stimulation, achieving optimal modiolar proximity in cases with short CDL should be considered in CI planning.

In our cohort, the majority of *SOX10*-related WS cases were classified as WS2, characterized by the absence of diagnostic clues beyond iris anomalies and hearing loss at a very young age. In addition, most cases were sporadic, with no apparent family history, making early clinical suspicion challenging. Given this context, the finding that 87.5% (7/8) of WS patients exhibited vestibular anomalies on imaging represents a critical diagnostic clue. Therefore, when evaluating pediatric patients with bilateral severe-to-profound hearing loss, the presence of iris anomalies and vestibular system abnormalities on imaging should raise suspicion for *SOX10*-related WS. Moreover, clinicians should anticipate the possibility of short CDL in these patients, which may have implications for electrode selection and CI planning.

The limitation of the present study lies in the potential unreliability of the ECA algorithm in cases of IP, particularly IP type I and IP type III, where the modiolar structure is either absent or poorly defined. Since the ECA algorithm, as employed in OTOPLAN, is based on a standard-shaped cochlea, its applicability in malformed cochlear structures remains problematic. This limitation underscores the need for alternative approaches such as TBCT-based direct three-dimensional segmentation of the cochlear outer wall or modifications to the algorithm when addressing cases with significant anatomical variations. Further research is required to refine computational models that can account for such structural anomalies, ensuring accurate assessments across diverse patient populations. Furthermore, while the present investigation was limited to more prevalent etiologic groups in which at least two cases of short CDL were identified to reduce the likelihood of chance findings, other etiologies in which only one case of short CDL was identified warrant future investigation.

## Conclusion

This study highlights significant CDL variations across different hearing loss etiologies, with the shortest CDL observed in *SOX10*-related WS cases, often with vestibular anomalies. Preoperative identification of short CDL aids in CI planning, thereby guiding electrode selection based on residual hearing or modiolar proximity.
